# Symbiotic interactions of plant microbiota in alleviating stress: a review

**DOI:** 10.3389/fpls.2026.1892395

**Published:** 2026-09-17

**Authors:** Shiva Sai Prasad, Atul Singh, Pramod Ramteke, Csilla Veres, Rita Büchner, Csaba Vágvölgyi

**Affiliations:** 1 Department of Agriculture, Koneru Lakshmaiah Education Foundation, Vijayawada, India; 2 Department of Molecular Biology and Genetic Engineering, Rashtrasant Tukadoji Maharaj (RTM) Nagpur University, Nagpur, India; 3 Department of Biotechnology, Hislop College, Nagpur, Nagpur, India; 4 Department of Biotechnology and Microbiology, Faculty of Science and Informatics, University of Szeged, Szeged, Hungary

**Keywords:** DBTL, holo-omics, mutualism, nano-microbiology, stress, symbiosis, synthetic microbes

## Abstract

Plants continuously encounter a wide range of biotic and abiotic stresses that adversely affect their growth, development, and productivity. Because they are sessile, plants cannot escape these unfavorable conditions and therefore rely on a diverse array of morpho-physiological, biochemical, and molecular adaptations to survive. Among these adaptive strategies, symbiotic associations with beneficial microorganisms have emerged as a crucial mechanism for enhancing stress tolerance. These plant–microbe interactions are mediated by intricate chemical signaling networks that regulate nutrient exchange, defense responses, and stress adaptation. Despite their immense potential for sustainable agriculture, the large-scale application of beneficial microbes remains limited owing to poor microbial establishment under field conditions and an incomplete understanding of the complex mechanisms governing plant–microbe mutualism. Deciphering these interactions is particularly challenging because they are highly dynamic and involve continuous communication between plants and diverse microbial communities. Recent advances in omics technologies, synthetic biology, and nanotechnology provide unprecedented opportunities to unravel these complex relationships at the molecular and systems levels. This review summarizes plant adaptive strategies under biotic and abiotic stresses, examines the role of microbial symbiosis in stress alleviation, and highlights emerging approaches, including multi-omics integration, synthetic microbial consortia, engineered quorum-sensing circuits, holobiont-level analyses, and nanoparticle-mediated modulation of the rhizosphere microbiome, for understanding and engineering beneficial plant–microbe interactions. Collectively, these advances offer new insights into symbiotic crosstalk and provide a foundation for developing resilient and sustainable agricultural systems.

## Highlights

- Plant root exudates shape and recruit beneficial microbiota
- Quorum sensing for biofilm formation
- Microbiota contribute to plant defense responses
- Synthetic microbes and nano-enabled approaches
- Omics approaches for understanding mutualism under stress

## 1 Introduction

Over the past century, there has been a drastic shift in the climate and seasons because of global warming. Globally, biotic and abiotic stresses significantly impact agricultural productivity, leading to substantial economic losses. Abiotic stresses, such as drought, salinity, and extreme temperatures, account for approximately 50% of global food crop losses, while biotic stresses, including diseases and pest infestations, contribute to around 30%. Similarly, this affects not only agricultural crops but has also been found in forest trees through deforestation. These stress can significantly impact plant growth, development, and productivity (Ait-El-Mokhtar et al., 2023). They can be classified into biotic and abiotic stress. A biotic stress is caused from living organisms, *viz.*, pests and diseases, while the abiotic includes external environmental conditions, *i.e.*, freezing, drought, salt, elevated temperature, toxicity, flood, *etc.* (Sena et al., 2024).

Stress can be overcome using modern techniques like drip irrigation, integrated pest and disease management, and the application of bio-stimulants. And among them, one of the economical and feasible solutions is the usage of biofertilizers (Chaudhary et al., 2022). They are alternatives to the chemical fertilizers, improving the soil health, productivity and yield. Also helpful in nutrient mobilization, solubilization, phytohormone production and stress management (Shahzad et al., 2025). Though they are potential, limited applications have been reported due to microbial survivability and a failure to understand mutualism. To overcome this barrier, modern biotechnological interventions need to be applied.

Similarly, plants have evolved various survival strategies to cope with these conditions. Includes anatomical modifications, biomolecule synthesis, and a symbiotic association with microorganisms (Rossi et al., 2017). Several studies have reported that, plants undergo several morphological changes under adverse climatic conditions (Khan et al., 2020a), changing the shape of stomata (Sadhukhan et al., 2022), modification of leaves (Fleta-Soriano and Munné-Bosch, 2016) and production of adventitious roots (Mergemann and Sauter, 2000). Further, plants also reported to synthesizes specific proteins (Batista‐Silva et al., 2019), photosynthates (Miras-Moreno et al., 2021; Nasrullah et al., 2022), enzymes (Ding et al., 2023; Meena et al., 2019) and metabolites (Liu et al., 2019; Zhang et al., 2023). These biomolecules, whose production is genetically regulated or influenced by microbial associations, help in osmotic adjustment and reduce oxidative damage. A symbiotic association between inter-kingdoms indicates a mutual understanding in exchange of resources (Cheng et al., 2021). These mutualisms have revealed a synergetic effect on the plant. Beneficial microbes have been found on phyllosphere (Zhang et al., 2023), rhizosphere (Yue et al., 2023) and endophytic (Sena et al., 2024) helping in improving nutrient availability and modulating stress responses. Although plant–microbiome interactions under stress have been extensively studied, most studies have focused on individual stress types or microbial taxa, and an integrated understanding remains limited. In this article, we will discuss the studies on synthetic microbial consortia design, holobiont-level multi-omics integration, engineered quorum-sensing circuits for host–microbe communication, and nanoparticle-mediated modulation of the rhizosphere microbiome. We further discuss the potential application of these approaches to genome-level CRISPR-based editing of microbiome-associated host loci and to microbial bioinoculant trials.

## 2 Plant adaptation strategies

Plants can adapt to harsh environmental conditions through several modifications. These modifications can help them survive with minimal damage. These adaptations can be classified into morpho-physiological, biochemical, and molecular (**Table 1**).

**Table 1:** Plant-microbiome interaction mechanisms in alleviating stress.

Adaptation strategy	Host	Microbe	Stress type	Mechanism	Reference
Biochemical and molecular	Sorghum	Bacillus subtilis	Flood	Increased nitrogen and chlorophyll	(Aquino et al., 2019)
Biochemical and molecular	Sorghum	Monoderm-enriched bacterial taxa	Drought	Drought-associated enrichment of monoderm taxa; altered metabolite exchange	(Xu et al., 2018; Xu and Coleman-Derr, 2019)
Biochemical and molecular	Citrus	Diversispora spurca (AMF)	Flood	Increased antioxidant enzyme activity	(Wu et al., 2013)
Biochemical and molecular	Rice	Endophytic fungi (e.g., Nigrospora oryzae)	Flood	Reduced ethylene (ACC deaminase) and oxidative damage	(Sodhi and Saxena, 2023)
Biochemical and molecular	Rice	Root-associated microbiome	Drought	Compartment-specific restructuring of root-associated microbiomes under drought	(Santos-Medellín et al., 2017)
Biochemical and molecular	Rice (assisted)	Cultivable cold-tolerant community (from pea rhizosphere)	Chilling	Improved rhizosphere nitrogen availability and plant N uptake; drives microbial succession	(Zhang et al., 2024)
Biochemical and molecular	Tomato	Pseudomonas spp. (endophytes)	Salt	ACC deaminase-mediated reduction of stress ethylene; growth promotion	(Ali et al., 2014)
Biochemical and molecular	Limonium sinense	Bacillus flexus	Salt	Improved K+/Na+ balance; increased proline and flavonoids	(Xiong et al., 2020)
Biotic (pathogen)	Pepper (Capsicum)	Rhizosphere bacteria (e.g., Pseudomonas spp.)	Aphids/pathogens	Rhizosphere recruitment and priming via SA/JA signaling	(Lee et al., 2012)
Biotic (pathogen)	Sugar beet	Proteobacteria and Actinobacteria (endosphere)	Pathogens	Enrichment of disease-suppressive functions; pathogen suppression	(Carrión et al., 2019)
Biotic (pathogen)	Wheat	Pseudomonas	Fusarium	Reduced fungal virulence via altered histone acetylation	(Chen et al., 2018)
Morpho-physiological	Angiosperms	Streptomyces (actinobacteria)	Drought	Increased root colonization and support of rhizosphere function under drought	(Xu et al., 2018)
Morpho-physiological	Dichanthelium lanuginosum	Curvularia protuberata	Heat	Symbiosis confers thermotolerance	(Redman et al., 2002)
Morpho-physiological	Various	Nitrogen-fixing symbionts	Drought	Improved plant performance under drought (case-dependent)	(Zhao et al., 2024)
Biotic (pathogen)	Tomato	Flavobacterium-enriched microbiome	Ralstonia wilt	Competition for resources; microbiome-mediated suppression	(Kwak et al., 2018)

### 2.1 Morpho-physiological adaptations

Morpho-physical changes are external structural modifications of existing structures and metabolic processes through internal changes, e.g., aerial roots, adventitious roots, osmolytes, Stomata closure, *etc.* Several morphological changes have been reported under salt stress in soybean (Barberon et al., 2016; Silva et al., 2021) and *Arabidopsis* (Bensussan et al., 2015). During stress, the plant reduced metaxylem tissue in roots to reduce cavitation. Further, plants increased the epidermis and endodermis layers to protect against salt damage. Under high temperatures and drought, plants primarily undergo the production of sclerenchyma tissues in the parenchymatous region (Falsini et al., 2022), also the initiation of the deep root system for better water uptake. However, under flood conditions, plants tend to synthesize high amounts of auxin and ethylene to produce adventitious roots, while the role of these may differ with plants (Jia et al., 2021).

### 2.2 Biochemical and molecular adaptations

Further, the biochemical and molecular factors refer to changes in the plant’s metabolic molecules, e.g., accumulation of antioxidants, synthesis of heat shock protein, *etc.* The adaptive changes include antioxidant accumulation, production of ROS and secondary metabolites, proteins, and enzyme synthesis. The primary damage caused during the stress will be from the accumulation of reactive oxygen species (ROS) produced by mitochondria, cytoplasm, peroxisomes, and photosystems to balance the cell’s energy functions (Kasote et al., 2015). In the above condition, antioxidants mitigate ROS by scavenging free radicals. Secondary metabolites, such as alkaloids, terpenoids, phenolics, etc., also carry out a similar scavenging function. These metabolites possess more aromatic rings and hydroxyl groups, which help conjugate the side chains of aromatic rings in scavenging.

## 3 Stresses and adaptation

### 3.1 Abiotic stress

Abiotic stress refers to the negative impact of non-living factors on living organisms in a specific environment, significantly affecting plant growth and agricultural productivity. Plants have developed complex mechanisms to cope with these stresses, involving physiological, biochemical, and molecular responses. These strategies evolved over the years in response to changes in climatic conditions. Plants respond to abiotic stress through a variety of complex mechanisms that enable them to survive and adapt to challenging environmental conditions. These responses include stress perception, signal transduction, gene expression regulation, physiological adjustments and symbiosis. Several findings suggest the interaction between species and kingdoms in the symbiotic association through histone acetylation. A recent finding suggests that a few beneficial microbes directly influence the rhizosphere by hindering pathogenic fungal protein production. *Pseudomonas piscium*, a beneficial microbe in the wheat rhizosphere, was found to inhibit a pathogenic microbe, Fusarium graminearum, by directly affecting the activity of protein production (Chen et al., 2018). As per the reports, there are several mechanisms involved in communication between the host and microbe, “Quorum sensing” a mechanism where bacteria send a signal (homoserine lactone) for cooperation with different taxa (Trivedi et al., 2020). This process is used by bacteria to coordinate their behavior in response to population density. Bacteria release signaling molecules called autoinducers, which accumulate in the rhizosphere as their populations grow. When a threshold concentration of these molecules is reached, it will trigger the changes in gene expression, allowing the bacteria to synchronize activities such as biofilm formation, virulence, and bioluminescence. This mechanism was found to be helpful in overcoming bacterial wilt by upregulating the PapR/NprX/Phr peptide-based QS pathway (Zhang et al., 2026). Recent reports have found that the bacteria coordinated among themselves through production of LuxI/LuxR-type acyl-homoserine lactones(AHL) in gram negative and different types of peptide in Gram-positive bacteria (Pal et al., 2026). The Lux proteins are transcription factors that bind to AHLs to promote bacterial colonization. While plants do not produce AHLs themselves, they can perceive and respond to these molecules, which can significantly impact plant growth, development, and stress responses. Further, root exudates also plays an important role in structuring the shape of rhizosphere, during the initial growth phase, primary metabolites such as organic and amino acids were produced, while upon the initiation of reproductive phase, there was a shift towards production of secondary metabolite (amines, benzene, phenolics) (Li J. et al., 2025).

#### 3.1.1 Flood adaptation strategies

One of the best adaptations is mangroves; their roots have been modified in specialized root system *pneumatophores* to breathe under submerged conditions (Kitaya et al., 2002). Similarly, several modifications have been reported to form adventitious roots, aerenchyma, hypocotyl, crown, brace, nodal, and stem roots under flood (Li et al., 2006; Vidoz et al., 2016), causing lower oxygen availability with high ethylene accumulation, leading to epinasty. Some plants have evolved strategies by producing adventitious roots, as reported in wheat (Li et al., 2023), Brinjal (Dawood et al., 2014; Zhang et al., 2017) and tomato (Vidoz et al., 2016). These phenomena are still more evolved in rice (Lin et al., 2023). The adventitious roots formed during the initial three days of flooding will be less thick than those formed after seven days. Flooding can also affect the symbiotic microbiota in the rhizosphere (fungi, bacteria, archaea, oomycetes, and viruses).

The number of microbes detrimental to plants has increased compared to the number of symbiotic microbes in wheat (Francioli et al., 2021). The detrimental microbes are adapted to anaerobic conditions. However, the phyllosphere microbiota in wheat influenced plant growth and development during the flood. The Proteobacteria and Actinobacteriota have increased the synthesis of nitrogen, chlorophyll content (Aquino et al., 2019) and auxin (Borah and Thakur, 2020). Root aerenchyma facilitates internal gas diffusion and oxygen delivery to submerged tissues, helping maintain root aeration and redox balance under flooding stress. The oxygen in the root tips will be lost through radial oxygen loss up to 1–3 mm diameter, helping the aerobic microbes to survive and protect the rhizosphere from the attack of detrimental microbes such as *Fusarium* sp and *Phytophthora* sp. Arbuscular mycorrhiza has been found promising under flood with the presence of aerenchyma tissue facilitating the gas exchange in rice (Martínez-Arias et al., 2022). The endophytic fungus also showed antioxidant potential, as demonstrated by DPPH (2,2-diphenyl-1-picrylhydrazyl) and ABTS 2,2′-azino-bis(3-ethylbenzothiazoline-6-sulfonic acid) radical-scavenging assays. Along with this, it was also found to be synthesizing “1-Aminocyclopropane-1-carboxylate (ACC) deaminase,” causing a drop in ethylene levels to promote vegetative growth (Sodhi and Saxena, 2023). Also, root morphology has been coevolved with Glomeromycota for nutrient exchange and interaction (Willis et al., 2013).Arbuscular mycorrhizal (AM) was reported to bridge the communication through its hyphal mycelial networks. The network helps in phosphorus/water acquisition (Aguilar-Trigueros and Frew, 2026), strigolactone-mediated signaling initiating symbiosis (Ge et al., 2022) and under multiple stresses (Khan et al., 2020b). Further, researchers can aim to bioengineer the microbe, as mentioned above, to establish a symbiotic relationship with commercial crops, which would help develop flood-tolerant crops.

#### 3.1.2 Drought and high temperature adapting strategies

Sometimes, plants experience prolonged exposure to water deficit, also known as drought. During drought stress, plants adopt water conservation measures, i.e., lateral roots for more absorption, increased leaf shedding, and reduced leaf size and numbers (Lamalakshmi Devi et al., 2017). These adaptations are reported in crops like Grapes (Gómez-del-Campo et al., 2003), Maize (Shao et al., 2021; Zhou et al., 2020), and Tobacco (Liu et al., 2023). Similarly, sclerophyll is another modification, and leaves are hardened to reduce moisture loss (Nardini, 2022). These anatomical adaptations have improved plants’ tolerance to drought. The thick cells help lower cell turgor pressure, leading to reduced water loss and translocation in the vascular pathway. Meanwhile, this could lead to leaf water deficit and kill the mesophyll cells. Phytohormones also contribute to stress tolerance; abscisic acid together with jasmonic acid can promote stomatal closure (Finkelstein, 2013; Mathur and Roy, 2021); ethylene can influence osmolyte accumulation (e.g., proline), contributing to osmotic adjustment (Wahab et al., 2022). Proline accumulation is often accompanied by changes in redox metabolites such as ascorbate and glutathione, which are important for thermotolerance, transpirational cooling, and membrane stability under high-temperature stress (Ul Hassan et al., 2022).

The studies have indicated the effect of endophytic actinobacteria in enhancing drought tolerance by producing thick-walled spores and supporting the rhizobacteria (Fitzpatrick et al., 2018). *Streptomyces albidoflavus* OsiLf-2, an endophytic actinomycete, produces osmolytes such as proline, polysaccharides, and ectoine, which enhance the osmotic adjustment ability of rice under drought conditions, resulting in increased proline content by 250.3% and soluble sugar by 20.9% (Niu et al., 2022). Further, *Pseudomonas argentinensis*, modulate root architecture and gene expression through the abscisic acid (ABA) pathway in grass, crucial for drought response. Similarly, plants tend to produce glycerol-3-phosphate (G3P) in the root zone to promote the beneficial monoderms (actinobacteria, chloroflexi, and firmicutes) population, which may contribute to the production of antibacterial compounds and to stress tolerance. At the same time, plants also inhibit the diderms (proteobacteria, Bacteroidetes, and acid bacteria) by releasing excess ROS (**Figure 1**) (Xu et al., 2018). Monoderm bacteria, characterized by thick cell walls and lack of an outer membrane. They are associated with altered plant metabolism and increased activity of bacterial ATP-binding cassette (ABC) transporter genes. These changes suggest that monoderm bacteria may help in optimizing nutrient uptake and stress tolerance. Though they are beneficial, practical application of monoderm is often limited by the low stability and activity of their enzymes (P450), requires specific conditions and cofactors to function effectively. So, future researchers can look for developing a stable monoderm to develop drought tolerance in commercial crops.

**Figure 1:**
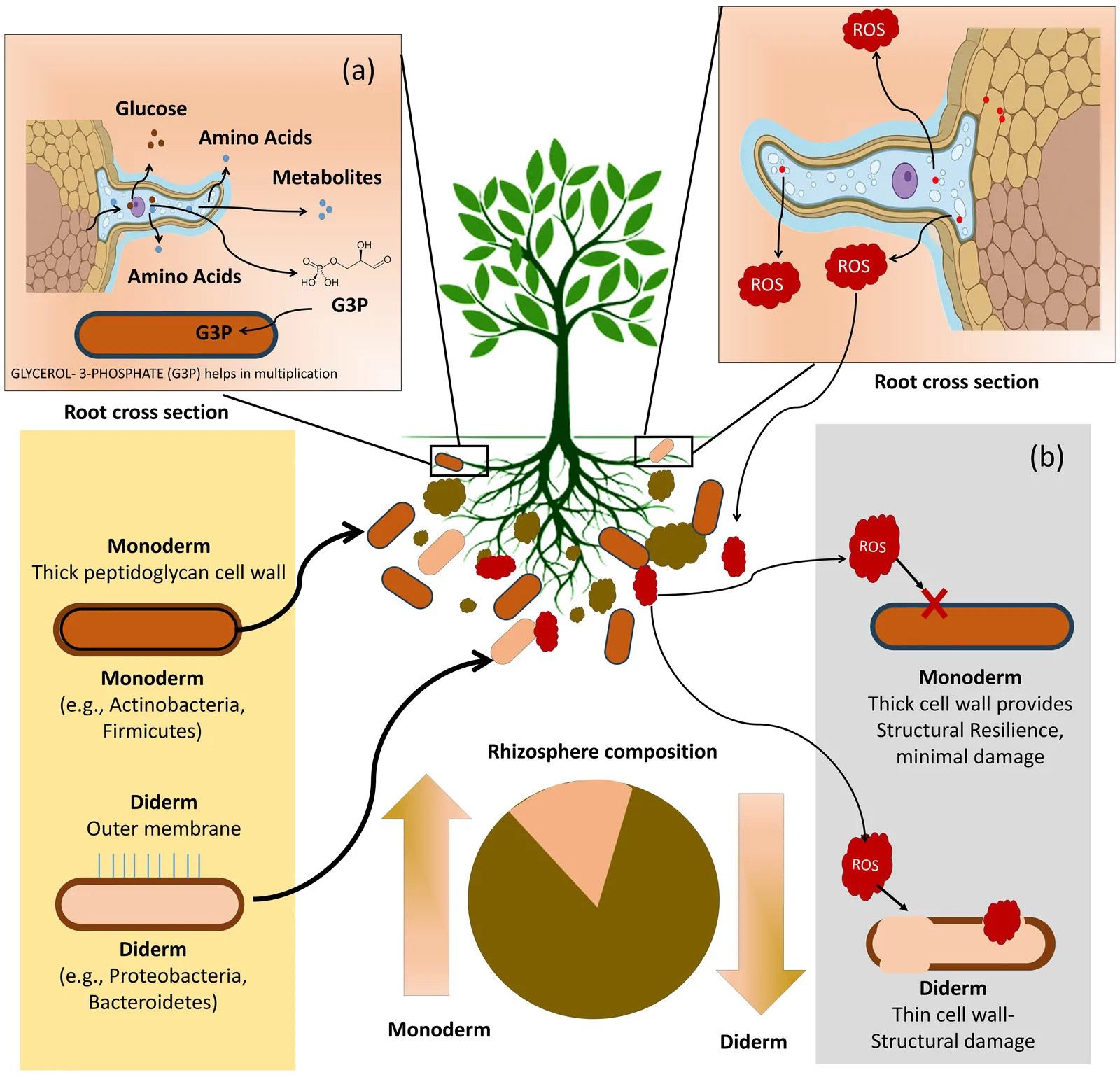
Conceptual model of drought-driven shifts in root metabolite exudation and selection of monoderm vs diderm taxa. **(a)** Plant exudates (including glycerol-3-phosphate, G3P) may support enrichment of some monoderm lineages; **(b)** oxidative stress and other selective pressures can disproportionately reduce diderm lineages. Note: monoderms typically have a thick peptidoglycan cell wall and lack an outer membrane, whereas diderms have an outer membrane and a thinner peptidoglycan layer.

#### 3.1.3 Freezing and chilling adapting strategies

Over time, plants have evolved with climate change for survival. As a result of evolution, they have developed tolerance to persisting climatic conditions. Highly evolved plants can be seen near-polar and desert regions, where few evolved to survive under shallow waters and few under high-temperature conditions, respectively (Nilsen and Orcutt, 1996; Thomashow, 1999). The regions having extremely low temperatures are known to be causing freezing (<0°C) and chilling (0–15°C) damage (Cen et al., 2016). However, some studies indicated modifications in changes in cell membrane composition, such as increased unsaturated fatty acids, maintain membrane fluidity at low temperatures (Ramakrishnan et al., 2022; Satyakam et al., 2022). And upregulation of cold-responsive (COR) genes, leading to the production of antifreeze proteins (AFPs) to inhibit crystal formation within plant tissues, stabilizing cellular structures and protection against ice formation to maintain cell integrity and permeability. Further, several plants also produce cryoprotectants to increase membrane integrity (Saddhe et al., 2021). These compounds help prevent the crystallization of cell organelles and balance osmotic adjustments. Evidence indicates that artificial rhizosphere regulation using cold region soils improves the tolerance to chilling injury. It involves manipulating the plant-soil interface to optimize plant growth, nutrient uptake, and soil health. This approach aims to enhance the beneficial interactions between plant roots and soil microorganisms, improving the nutrient availability, and mitigating the environmental stresses. This was practically demonstrated when pea rhizosphere soil was extracted from a cold region and used for successful cultivation of rice under chilling conditions (Zhang et al., 2024). This artificial rhizosphere regulation can be easily applied to crop plants to develop tolerance to chilling and freezing temperatures.

Microbes also play a crucial role in chilling and freezing injury tolerance. *Arthrobacter*, *Flavimonas*, and *Pseudomonas*, have been shown to induce chilling resistance in tomato (Subramanian et al., 2016). These bacteria enhance plant growth and reduce membrane damage by activating antioxidant enzymes and promoting proline synthesis under chilling stress conditions. In lettuce, root morphology change was significantly observed under chilling conditions, with specific bacterial families like *Oxalobacteraceae* and *Pseudomonadaceae*. Although direct growth-promoting effects were not observed in lettuce, related strains from grass roots demonstrated potential for promoting growth under cold conditions (Persyn et al., 2024). Similarly in Arabidopsis, an endophytic fungus *Piriformospora indica* enhanced the freezing tolerance by stimulating the expression of CBF genes, which are crucial for cold acclimation and stress response (Dastogeer et al., 2022). By analyzing such processes, it helps to understand how plants precisely modulate root exudates to either recruit or repel specific microorganisms, thereby shaping their colonization patterns.

#### 3.1.4 Salt stress adaptations

Salinity is one of the significant constraints driven by human activities (Acosta-Motos et al., 2017). The stress condition develops over a period through continuous irrigation of unsuitable groundwater and excessive application of chemicals to the soil. The major ions are sodium and chloride; the excessive presence of these salts in the soil causes difficulty in plant water uptake and reduced conductivity for the uptake of other ions by the plant (Cha et al., 2021). To overcome the effect of salts, plants have developed mechanisms to alter the compartmentalization of sodium in root cells from cytosol to vacuole through Sodium/hydrogen antiport for maintaining the osmotic balance while driving the water (Zhang and Blumwald, 2001). Similarly, antioxidants and growth hormones are significant in keeping osmotic adjustments and scavenging ROS (Reactive oxygen species).

The damage caused by the overaccumulation of salts, expelled from the cytosol via Na^+^ efflux through the plasma membrane via the Na+/H+ antiporter salt overly sensitive 1 (SOS1) pathway. This helps the plants to regulate the osmotic adjustment and vacuolar sequestration (Xie et al., 2022). A similar salt tolerance response was reported in lettuce (Sardar et al., 2023), chickpeas (Ahmad et al., 2016), and brassica (Shah et al., 2021). The endophytic microbes *Pseudomonas* sp. and *P. migulae* were found to reduce the ethylene effect under salt stress through the synthesis of ACC deaminase while promoting nitrogen fixation in tomatoes (Ali et al., 2014; Hardoim et al., 2008). The study on Limonium under salt stress indicated a positive symbiotic association between the plant and *Bacillus flexus.* As the plant is a halophyte, the root zone was detected to secrete organic acids, i.e., palmitic, 2-methylbutyric, palmitoleic, stearic, and Eugenol. These acids help move selected microbes to colonize in the root zone. Similarly, the microbe has induced higher production of proline and flavonoids to reduce the ROS damage and also helped in K^+^/Na^+^ ion balance in the plant (Xiong et al., 2020). This information can helps to understand the cross-talks between hosts and microbes in alleviating salt stress and can be extended to similar genotypes in the future (**Figure 2**).

**Figure 2:**
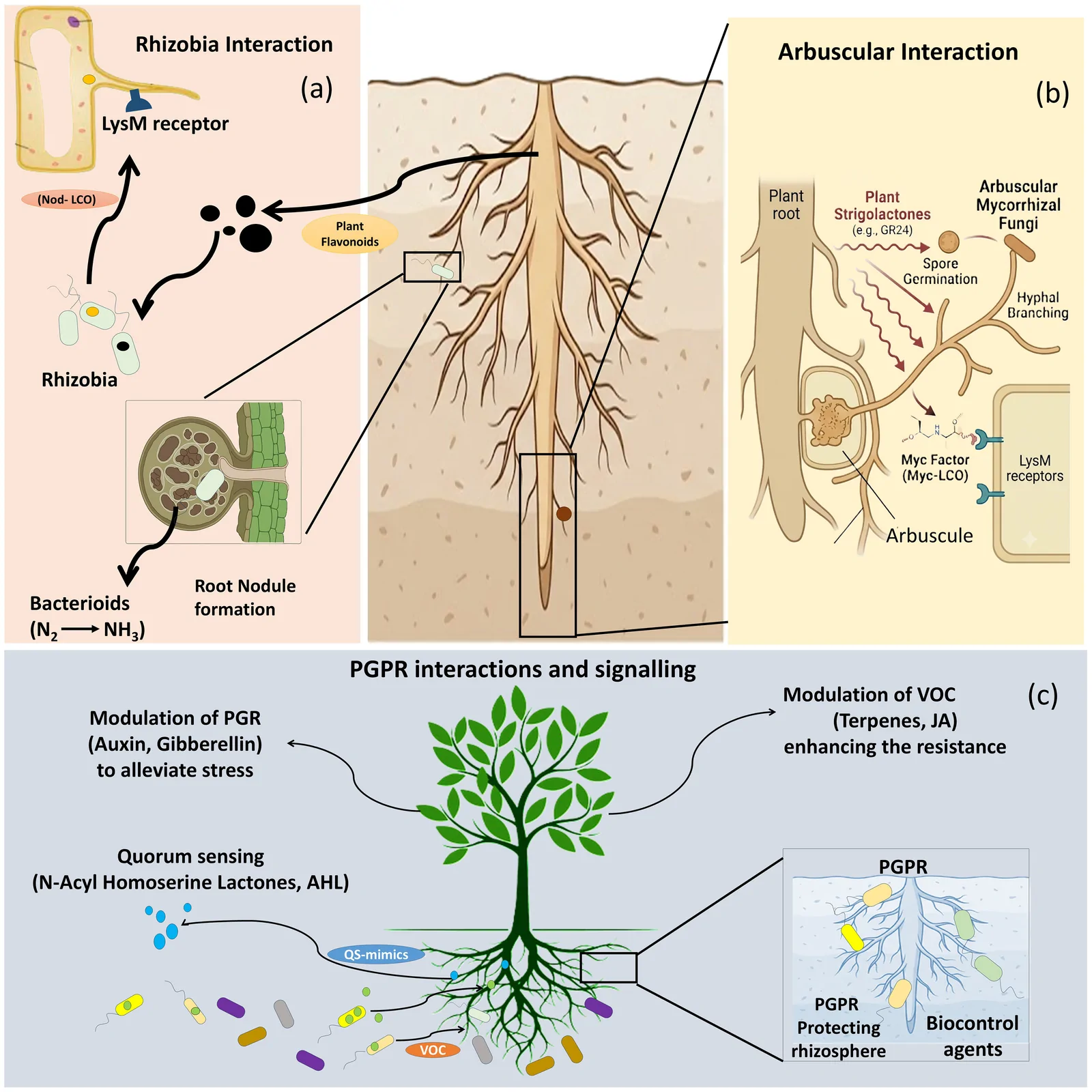
Signaling and crosstalk between plants and microbiota: **(a)** rhizobia interact with plants via Nod factors (Nod-LCOs) and plant flavonoids; **(b)** arbuscular mycorrhizae interact via Myc factors (Myc-LCOs) and plant strigolactones; **(c)** plant growth-promoting rhizobacteria (PGPR) can use quorum-sensing signals and other metabolites to influence plant volatile organic compounds and defense responses.

### 3.2 Biotic

Biotic stress occurs during favorable seasons or when a pest or disease outbursts due to the availability of favorable conditions. These epidemic or endemic conditions cause significant economic loss to the plants and their productivity (Jha and Mohamed, 2022).

#### 3.2.1 Tolerance to diseases

Plants have developed tolerance towards significant biotic stress through physiological and molecular modifications during evolution. Some proteins have been discovered in plants as a self-defense mechanism against pathogens. One of the recent molecules discovered, “Osmotin”- multifunctional protein that plays a significant role in enhancing disease resistance in plants, acts against fungal pathogens by rupturing the cell walls of fungi through intercellular signal transduction and activation of protein kinase (Bashir et al., 2020). A similar self-defense mechanism was also recorded in barley infected with *Fusarium* sp.*;* the plant produced t-cinnamic acid through roots using *de novo synthesis* as self-defense (Lanoue et al., 2010). This self-defense is also known as induced systemic resistance (ISR) or Systemic acquired resistance (SAR). It is a long-lasting protection against a wide range of pathogens, involving a complex interplay of chemical signals and pathways that coordinate to enhance the plant’s immune response. Salicylic acid (SA) is a crucial signaling molecule in SAR, transducing the signal through NPR1, a nuclear-localized protein that interacts with transcription factors to regulate gene expression (Li M. et al., 2025). Methyl salicylate, a derivative of SA, and lipid-based mobile signals are produced at the site of infection and travel through the phloem to initiate systemic protection in uninoculated tissues (Ezzat et al., 2014). Further, SAR response begins upon the lipid-based mobile signals with the recognition of plant resistance (R) genes and corresponding avirulence (avr) genes from the pathogen.

The pathogen resistance was also enhanced by the beneficial bacteria. In pepper (*Capsicum annuum*), foliar aphid infestation was shown to prime systemic defense responses involving salicylic acid and jasmonic acid signaling and to increase the population of beneficial *Bacillus subtilis* in the rhizosphere (Lee et al., 2012). It activated defense-related genes such as CaPR9, CaCHI2, SAR8.2, and CaLOX1. Further studies revealed that a similar infestation of biotrophic pathogen leads to the promotion of specific microbes in the rhizosphere of Arabidopsis for defense. These microbes form a biofilm in the root zone to protect the plant from detrimental pathogens (Berendsen et al., 2018). The sugar beet plants were enriched in the root endosphere with proteobacteria and actinobacteria, which helped to degrade pathogens’ cell walls (Carrión et al., 2019). In tomato, the enriched flavobacterium microbiome inhibited the infestation (wilt) of *Ralstonia* sp. by utilizing all available sugars to which the pathogen lectin binds for multiplication (Kwak et al., 2018). *Pseudomonas*, inhibited the growth of pathogenic blight fungi by hampering the synthesis of fungal protein activity (histone acetyltransferase) and antifungal compounds. The symbiosis between the rhizobia-legume follows a common signal transduction pathway for nitrogen fixation (Hirsch and Kapulnik, 1998). Further, it was also found that the rhizobia-legume symbiosis can occur without a Nod factor through secretion of rhizobia type III system, enhancing the plant immunity (Gourion et al., 2015). Many of the microbes have a probiotic effect on the plant in the suppression of diseases, and further studies can be taken to study the relationship between new strains and crops.

#### 3.2.2 Tolerance to pests and insects

Plants are immobile, making them vulnerable to several herbivores, pests, and insects. Pests have a specific plant selection habit that causes primary damage and economic loss. However, several plants have evolved herbivore resistance, producing herbivore-induced plant volatiles to attract predators. These volatiles contain (E)—β-caryophyllene, which is lethal to bacteria, and indole, which is toxic to caterpillars (Turlings and Erb, 2018).

Plants tend to produce volatile organic compounds whenever a plant is damaged by a herbivore (animal, insect, or pest). These compounds (terpenes, methyl jasmonate, and methyl salicylate) are emitted by plants in response to stress and serve multiple functions such as direct deterrence of herbivores and signaling to neighboring plants to prime their defenses. Several microbes also support this activity by inducing high concentrations of jasmonic acid (Ballaré, 2011). In pepper (*Capsicum annuum*), above ground whitefly infestation was shown to reshape the root-associated microbiota, illustrating the complex interactions among herbivorous insects, host plants, and below ground microbial communities (Kong et al., 2016). While the role of root-associated microbes in plant-insect interactions is well-documented, there are still gaps in understanding the full extent of these interactions, particularly concerning the indirect effects on herbivore natural enemies. Future research should focus on the complex networks of interactions involving multiple microbial species and herbivores to better understand their ecological and practical implications. Further in-depth studies can help us understand and develop a strong tolerance for biotic stress.

## 4 Advanced tools and techniques for elucidating plant–microbiome interactions

A comprehensive understanding of microbial interactions at both molecular and physiological levels can be achieved using advanced techniques (**Table 2**).

**Table 2:** Recent advanced techniques for understanding the symbiotic association of plant-microbiome.

Technique	Host	Microbe	Interaction/readout	Reference
GWAS	Sorghum	Rhizosphere community	Host loci influence rhizosphere microbiome composition	(Deng et al., 2021)
GWAS	Arabidopsis thaliana	Pseudomonas syringae (pathosystem)	Host genetic variation associated with resistance and microbiome outcomes	(Bartoli and Roux, 2017)
GWAS	Maize	Leaf microbiome (e.g., Methylobacterium)	Quantitative genetics of leaf microbiome traits	(Wallace et al., 2018)
GWAS	Tomato	Arbuscular mycorrhizae	Host loci regulate strigolactone biosynthesis and symbiosis	(Ge et al., 2022)
MWAS	Foxtail millet	Rhizosphere microbiome	Genotype-dependent microbial effects under stress; linked host genes	(Wang et al., 2022)
mGWAS	Tea	Sphingomonas	Metabolite-gene associations linked to microbiome assembly	(Tan et al., 2022)
Holo-omics	Various	Host + microbiome multi-omics	Integrate host and microbiome omics to infer functional interactions in a holobiont	(Kobel et al., 2024)

### 4.1 Omics

The omics involves understanding multiple approaches such as genome (genes), transcriptome (transcription), proteome (protein), and metabolomics (metabolites). The omics approach is essential to understanding the cross talks, while “meta” implies studying the entire set of samples (Nyholm et al., 2020). The host-microbe interaction starts with communication through the production of attractive molecules. These attractive molecules include nutrients, sugars, amino acids*, etc.* (Brencic and Winans, 2005). Further, plants also possess a particular binding site called “lectins” for interaction with the microbe. These lectins are non-catalytic and reversibly bind to mono and oligosaccharides (Naithani et al., 2021). Several microbes have been found to synthesize binding chemicals, such as cyclic glucan, through specific genes that help them bind to lectins in communication with plants (Jain et al., 2022; Konozy et al., 2022; Wanke et al., 2021). A new concept of a holo-omic approach has been discussed, which involves incorporating host and microbe data at multiple omic levels. This technique can combine host-microbe interaction datasets into biological signals through orthogonal methodologies. Generally, the technologies involve complex mechanisms using cluster analysis with functional enriching of biological functions to interact between host-microbiome using organic compounds (Kobel et al., 2024).

“Genome-wide association study (GWAS)” is a technique that helps to understand the variations present in the locus through genotypic and phenotypic association mapping. These variations may occur in the plant symbiosis with microbes to communicate (Beilsmith et al., 2019). Recently, phyllosphere DNA sequencing helped model microbial lineage with host-level interactions. The sorghum plant was found to have a specialized locus on chromosomes 4 and 6 for synthesizing disease resistance protein, which influences the rhizosphere microbe restructuring (Deng et al., 2021). Similarly, the “mGWAS (Microbiome-wide association studies)” technique indicated the role of several genes in the assembling of rhizosphere microbiota under stress (Wang et al., 2022). This tool was helpful under small sampling techniques for identifying several significant disease resistance with the help of microbial association. As per the reports, two genes have been identified: “microbiome-shaping (Ms) genes and microbiome-responsive (Mr) genes” as a symbiotic response (Lv et al., 2024). Further, the “mGWAS” technique is a multidimensional approach used to identify the role of metabolites and genes in symbiotic association (Fang and Luo, 2019). This tool helped to understand the role of *Sphingomonas* in Tea plants, developing resistance through synthesizing the β-carotene hydroxylase two genes as a secondary metabolite under specific conditions (Tan et al., 2022). The above techniques help understand the role of particular genes, metabolites, or mechanisms under symbiotic association. Modern web-based tools (Metaproteome, SECIM tool, and MetaLab) have been implemented, making an easy pipeline for automatic analysis (Starr et al., 2018). Host-microbiome interactions are highly non-linear; a genetic variant might influence diet preference, which alters the microbiome, rather than directly controlling the microbe itself. We currently lack the high-throughput functional validation pipelines needed to verify thousands of statistical mGWAS datasets.

Next-generation sequencing (NGS) technology helps detect and analyze the DNA sequence through high throughput and cost-effectiveness. This technique helps to sequence more DNA in less time and is cost-effective. Next-generation sequencing (NGS) enables high-throughput DNA and RNA sequencing and is widely applied in genomics, metagenomics, and transcriptomics. Proteomic and metabolomic analyses require complementary analytical platforms and can be integrated with sequencing-based approaches to provide a multi-omics view of plant–microbe interactions. Several studies indicate the role of NGS in understanding the role of plant-microbe interactions. Several sequencing platforms are available, such as Illumina/Solexa Sequencing, Roche/454 Pyrosequencing, Ion Torrent Sequencing, PacBio Sequencing, and Oxford Nanopore Sequencing. Among these sequencing, metabolomics has been a reliable tool for understanding the dynamic metabolite changes during the plant-microbe interaction. Further, fluxomics is also an advanced approach to understanding the interconversion of metabolites within a biological system (Xu and Fan, 2024). It helps to connect the omics with the plant’s phenotype through understanding the metabolic pathways. These techniques will help understand the genes-proteins-metabolites involved in crosstalks.

### 4.2 Synthetic microbes

Synthetic biology involves developing or modifying an organism’s biological process through genetic engineering to harness its benefits. In plant and microbe interactions, communication is more important to process the input to output; the input signal is transmitted from the sender, and a receiver receives the output signal. This has already been reported in barley (sender) and *Azorhizobium caulinodans* (receiver). As per the synthetic study, short-chain acyl-HSL changes the root morphology, and long-chain acyl-HSL improves immunity and defense (Babenko et al., 2022). Design-built-test-learn (DBTL) is an innovative technique used to build and test mechanisms (**Figure 3**) (Prasad, 2024). A logical communication system was designed to study the interaction between plants and microbes through DBTL cycles. The plants were included with *p*-coumaroyl-homoserine lactone (pC-HSL) through *Agrobacterium tumefaciens* as a receiver. Different bacterial strains were used as senders to couple environmental sensing to pC-HSL production. As per the results, the plant receiver showed up to ~39-fold induction in sterile soil compared with wild-type bacteria (Boo et al., 2024). Similar research can also be used to understand the signaling and sensing of signals under mutualism. Though synthetic engineering can design elegant, logic-gated therapeutic microbes, these engineered strains are often quickly outcompeted and eliminated by the resident microbiota.

**Figure 3:**
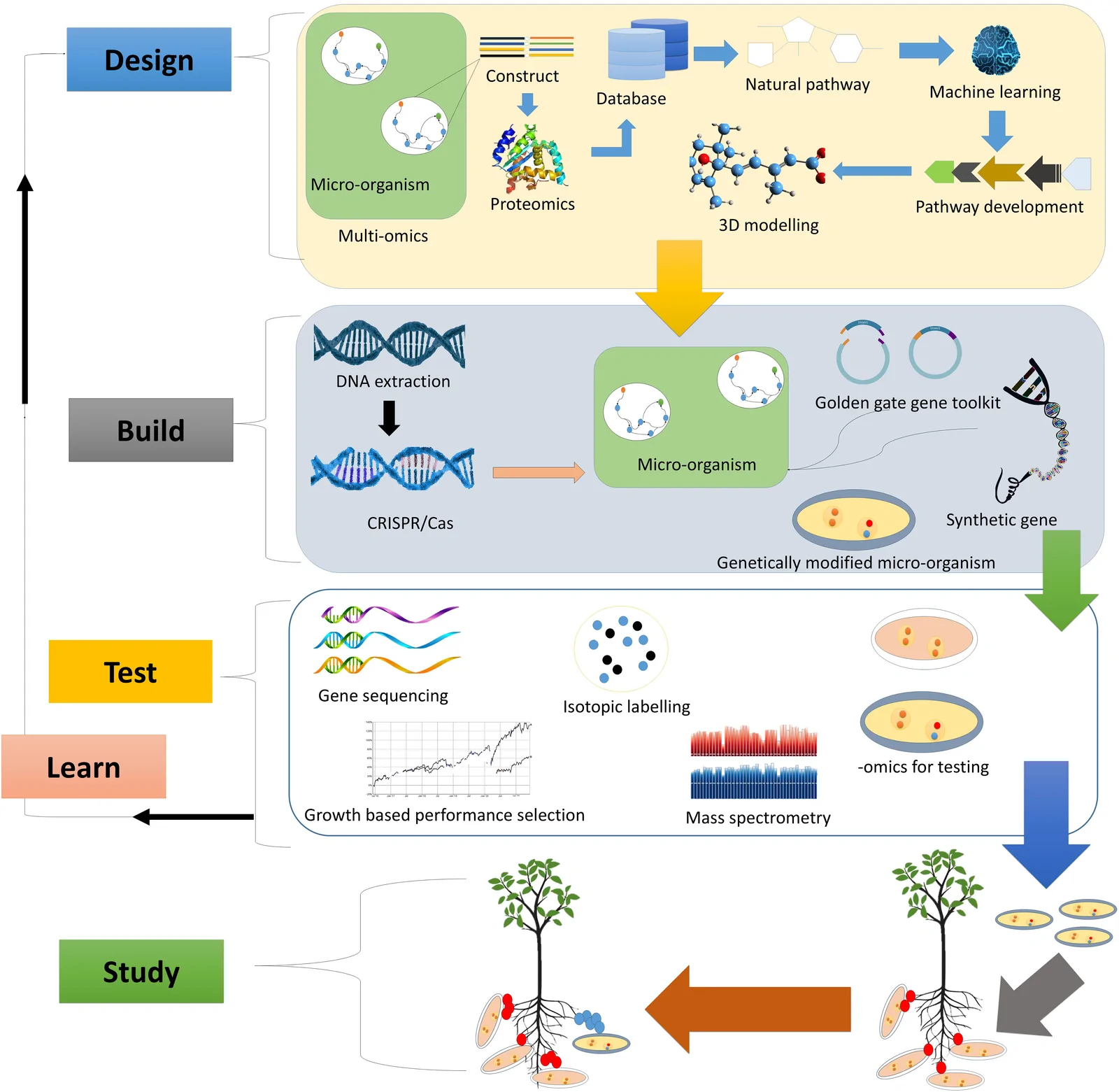
Design-build-test-learn (DBTL) cycle for engineering microbes to study and modulate plant-microbiome communication. The Design stage identifies candidate proteins/genes using multi-omics, database construction, machine learning, and 3D modeling; the Build stage generates engineered strains by introducing constructs into microbes; the Test stage evaluates performance using sequencing, isotope labeling, mass spectrometry, and omics; and the Learn stage uses the resulting data to refine subsequent designs and evaluate engineered consortia in controlled experiments.

### 4.3 Nano-microbiology

Nanotechnology is increasingly integrated into agriculture to enhance plant growth and development. Several studies have been conducted to understand the interaction of plant microbiome using nanoparticles (Sambangi et al., 2023). Nanobiotechnology can also help understand crosstalks through the engineering of the microbiome. The silver nanoparticles (AgNP) are helpful in understanding the rhizosphere of wheat microbiome. As per the reports, AgNPs with smaller size and a positive surface charge increased potentially harmful shifts in the microbiome, whereas larger AgNPs increased the abundance of beneficial, plant growth-promoting taxa (Przemieniecki et al., 2024). However, the inconsistent contributions of individual root-exudate metabolites to monoderm enrichment under drought caution against over interpreting these relationships as evidence of single-metabolite causation. Similarly, the Copper-tolerant bacterial population increased with the increased utilization of carbon sources under the application of nano-copper-based particles (Peixoto et al., 2024). Utilizing nanoparticles for targeted antimicrobial action holds massive promise. However, our understanding of how nanomaterials interact with complex microbial biofilms is still in its development.

Despite rapid advancements in genomic sequencing, critical knowledge gaps continue to bottleneck our understanding of plant-microbe interactions. At the molecular level, we have yet to fully decode the complex chemical language of root exudates that plants use to selectively recruit beneficial microbes, nor do we understand the rapid shoot-to-root systemic signaling pathways that coordinate this recruitment under stress. This lack of mechanistic clarity is compounded by a persistent lab-to-field translation gap, where introduced beneficial microbes, highly successful in sterile greenhouses, fail to colonize or survive when cultured under field conditions. Bridging these gaps through integrated multi-omics (metagenomics, metatranscriptomics, and metabolomics) is the critical next step required to move past descriptive taxonomic catalogs and toward predictably engineering the rhizosphere for climate-resilient, sustainable agriculture.

## 5 Conclusion

The mutualistic relationship between flora and their associated microbiota constitutes a fundamental aspect of plant vitality and robustness. The mechanisms through which microbiota alleviate stress operate through multifaceted pathways. They include modulation of antioxidant enzymes such as superoxide dismutase (SOD) and catalase, which neutralize reactive oxygen species (ROS) generated under stress conditions, modulate plant hormonal pathways, and enhance nutrient uptake. To achieve the above, we need to overcome the bottlenecks to effective practical application. Primarily, the limited reproducibility of laboratory/greenhouse results under field conditions is due to variable soil/climatic conditions, which require thorough field trials. Similarly, regulatory hurdles to the release of engineered/synthetic microbial consortia, including differing biosafety and GMO release frameworks, need to be eased. And large-scale formulation challenges for microbial bioinoculants with cost-effectiveness barriers have to be studied. Finally, a standardized efficacy metric and long-term ecological monitoring are required before nano-enabled or synthetic-biology-based approaches can be safely and effectively deployed at scale. A comprehensive understanding of these interactions at both the molecular and physiological levels can be achieved by integrating advanced techniques such as Omics, DBTL, synthetic microbes, and nano-enabled approaches within a unified framework. The knowledge may help to develop climate-resilient crops for adverse conditions to meet the food demand.
